# Scheduled feeding restores memory and modulates c-Fos expression in the suprachiasmatic nucleus and septohippocampal complex

**DOI:** 10.1038/s41598-017-06963-w

**Published:** 2017-07-28

**Authors:** Norman F. Ruby, Nathan Fisher, Danica F. Patton, Matthew J. Paul, Fabian Fernandez, H. Craig Heller

**Affiliations:** 1 Biology Department, Stanford University, Stanford, CA 94305 USA; 2 Department of Psychology, University at Buffalo, SUNY, Buffalo, NY 14260 USA; 3 Departments of Psychology and Neurology, BIO5 Institute, and The Evelyn F. McKnight Brain Institute, University of Arizona, Tucson, AZ 85724 USA

## Abstract

Disruptions in circadian timing impair spatial memory in humans and rodents. Circadian-arrhythmic Siberian hamsters (*Phodopus sungorus*) exhibit substantial deficits in spatial working memory as assessed by a spontaneous alternation (SA) task. The present study found that daily scheduled feeding rescued spatial memory deficits in these arrhythmic animals. Improvements in memory persisted for at least 3 weeks after the arrhythmic hamsters were switched back to *ad libitum* feeding. During *ad libitum* feeding, locomotor activity resumed its arrhythmic state, but performance on the SA task varied across the day with a peak in daily performance that corresponded to the previous daily window of food anticipation. At the end of scheduled feeding, c-Fos brain mapping revealed differential gene expression in entrained versus arrhythmic hamsters in the suprachiasmatic nucleus (SCN) that paralleled changes in the medial septum and hippocampus, but not in other neural structures. These data show that scheduled feeding can improve cognitive performance when SCN timing has been compromised, possibly by coordinating activity in the SCN and septohippocampal pathway.

## Introduction

Chronic memory impairments can develop after circadian timing is disrupted by ageing or shift-work^1^. These circadian disturbances have been correlated to problems with executive function and a higher probability of dementia onset^2, 3^. Several authors have suggested that memory impairments arising from circadian dysfunction of the light-entrainable oscillator (LEO) in the suprachiasmatic nucleus (SCN) might be improved by stimulating the circadian food-entrainable oscillator (FEO) that synchronizes to daily feeding schedules^4, 5^. We tested this idea in the Siberian hamster (*Phodopus sungorus*) because in this species, circadian timing in the SCN can be easily disabled without genetic, pharmacological, or surgical interventions^6^. The loss of circadian timing by this procedure results in substantial deficits in spatial and recognition memory. These memory deficits seem to be specific to circadian disruption and not due to sleep disturbances^7^. Thus, this model allowed us to assess the impact of the FEO on memory in the absence of the LEO without the confound of sleep disruption.

**Figure 1:**
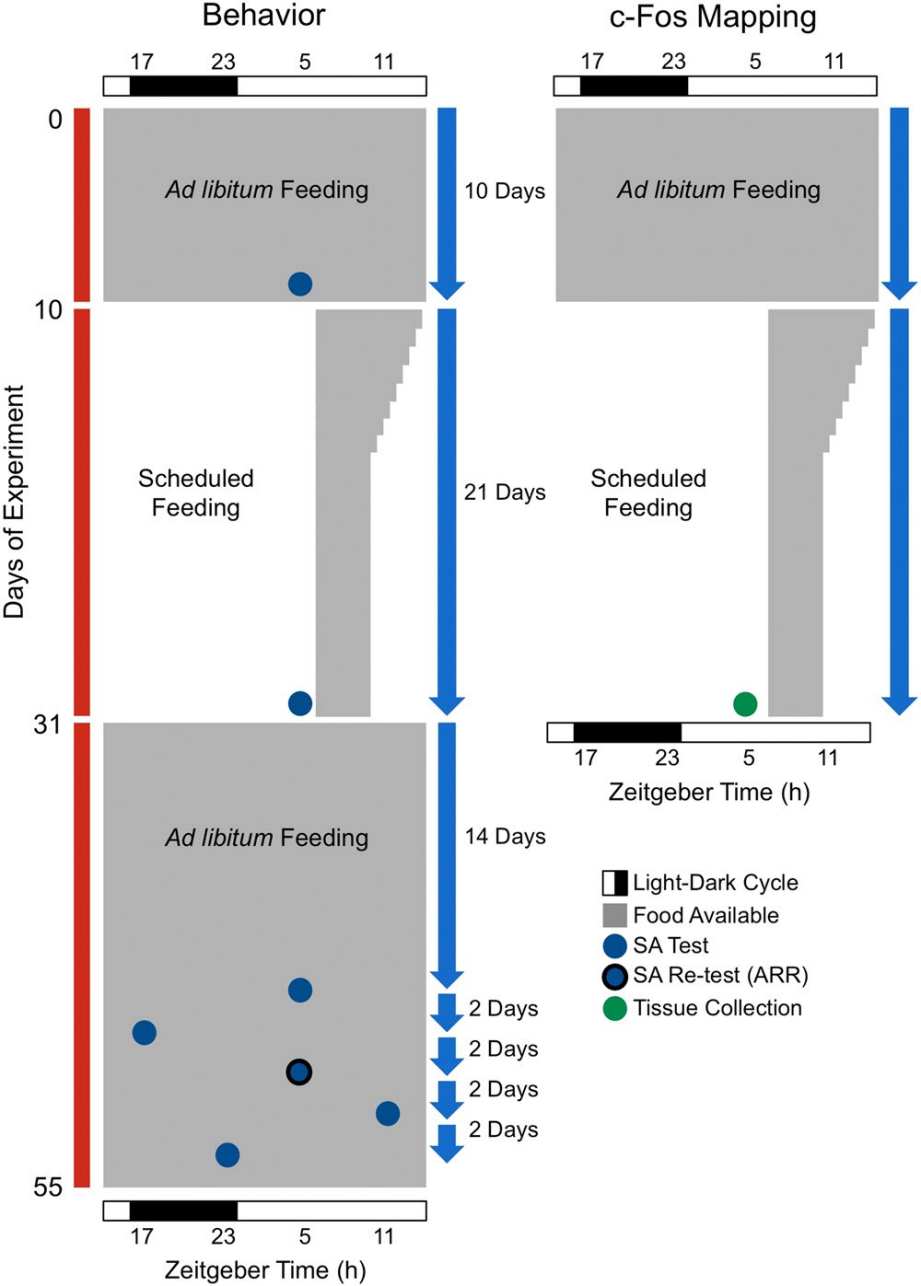
Schematic of behavior (left) and c-Fos (right) experiments. Time of day is indicated by the black and white rectangles. Room lights were on from ZT0-16. Memory tests were performed at four zeitgeber times (ZT; 17, 23, 5, 11). ZT17 and 23 indicate early and late night, respectively. The timeline for the project runs from top to bottom (red). The number of days for each section of the study is indicated by arrows (blue). The periods of scheduled feeding (SF) and *ad libitum* feeding are shaded in gray. SF began at ZT5 and was 8 h long on the first day, and then decreased by 30 min on successive days until it was 4 h in duration. SF lasted 21 days. The days on which memory was tested, as well as the times of day testing occurred, are indicated by filled circles (blue). Those blue circles show that memory was tested in the same group of animals at multiple time points with 2 days in between each test. Memory was tested twice at ZT5 in DPS-arrhythmic (ARR) animals as indicated by the blue circle outlined in black. The day and time of tissue collection for c-Fos studies is indicated by a filled circle (green).

In the present study, Siberian hamsters were exposed to a disruptive phase shift (DPS) protocol to permanently eliminate circadian rhythms in behavior and in the SCN^6, 8^. After baseline memory testing, separate groups of arrhythmic hamsters were either placed on a 21-day regimen of daily scheduled feeding (SF) or allowed to continue feeding *ad libitum*. Two groups of entrained animals were treated similarly to serve as controls. During SF, food was presented each day beginning 5 h after lights-on (i.e., zeitgeber time 5; ZT5). On the first day of SF, food was available for 8 h. On subsequent days, the window of food availability was reduced by 30 min each day down to 4 h (i.e., ZT5-9). This gradual conditioning gave the animals an opportunity to acclimate to the SF regimen. After 21 days of SF, animals were allowed 14 days of *ad libitum* feeding. After those 14 days, we began retesting the animals to show that memory improvements were not dependent on the continued presence of the feeding schedule or on any potential metabolic changes caused by SF (Fig. 1).

Spatial working memory was quantified by using a test of spontaneous alternation (SA) behavior. We chose this test because we previously hypothesized that an arrhythmic SCN could interfere with memory by altering activity in the septohippocampal pathway^6^. SA is critically dependent on this pathway and provides an accurate biometric readout of septohippocampal function (see Supplementary Materials for a detailed discussion on the history and neurobiology of SA). Unlike other hippocampal-dependent tasks, SA: 1) allows for frequent re-testing of individual animals without decrements in performance, 2) avoids introducing a competing arousal signal such as foot shock or using a food reward that would interfere with the establishment of food entrained memory rhythms^9^, and 3) does not require daily training sessions that might stimulate a daily rhythm of their own in arrhythmic hamsters.

## Results

### FAA is more robust in DPS hamsters

**Figure 2:**
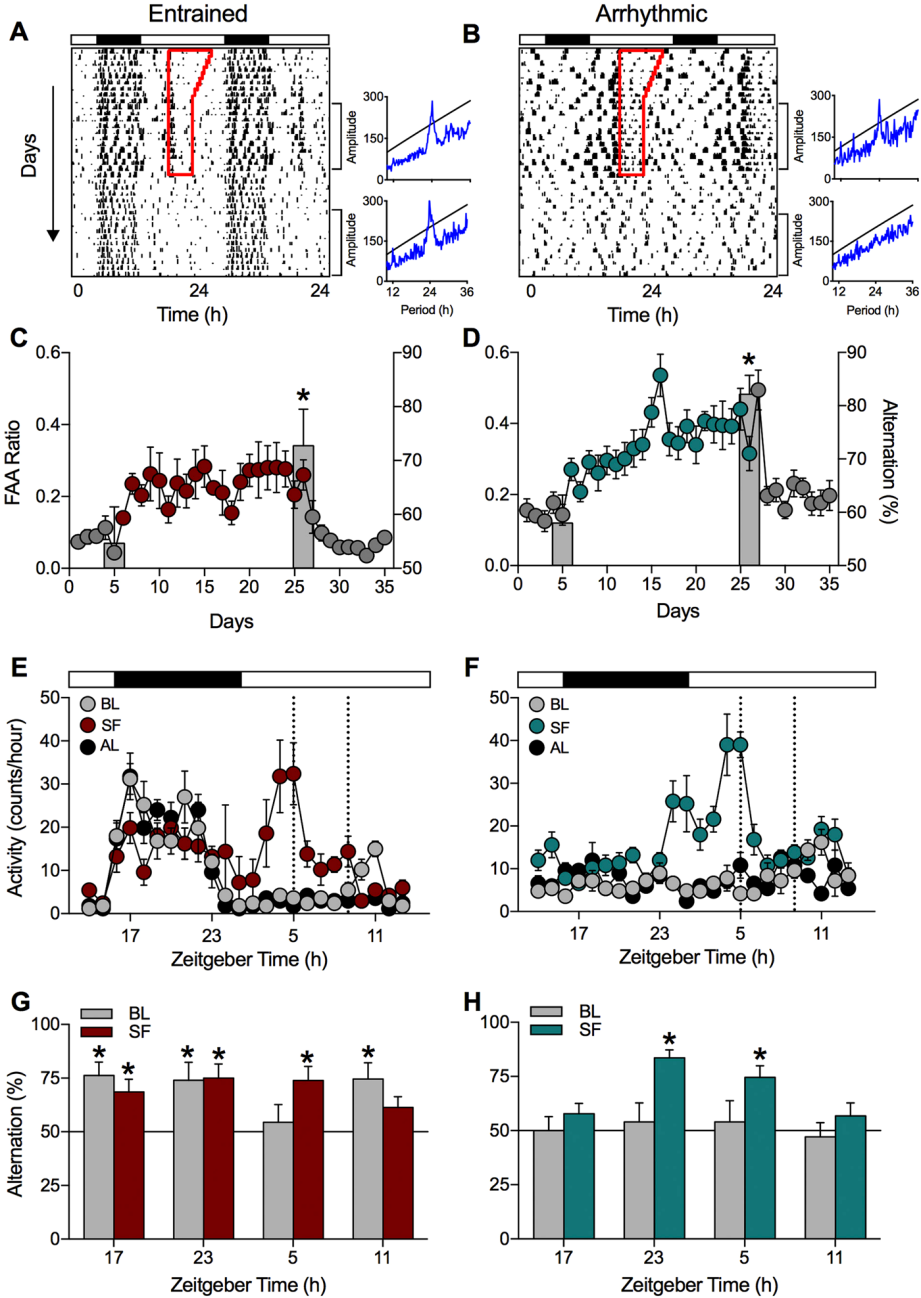
Scheduled feeding rescued spatial working memory in circadian arrhythmic hamsters and phase-delayed the rhythm of test performance in entrained animals. (**A**,**B**) Time of food availability (red outline) increased food anticipatory activity (FAA) prior to the time of feeding in both entrained (n = 9) and arrhythmic animals (n = 12). Activity returned to baseline levels when animals resumed *ad libitum* feeding. Brackets indicate portions of the actograms subjected to periodogram analysis. Peaks in blue above the black lines indicate statistically significant rhythms as determined by chi-square periodogram analysis. (**C**,**D**) The mean (±SE) daily FAA ratio was calculated during SF (red, green circles) and *ad libitum* feeding (gray circles). Gray bars represent the alternation scores (right y-axis) during baseline and on day 21 of SF. *Indicates significantly different from chance (i.e., 50%); P < 0.01. (**E**,**F**) Waveforms for total (mean ± SE) hourly locomotor activity during each phase of the study (BL = baseline, SF = scheduled feeding, AL = ad libitum) were constructed with the last 5 days of each condition (i.e., the last 5 days in panels C,D during baseline, SF, and AL). Data for each hour were averaged and plotted at the end of that hour (e.g., ZT17 is the mean of data from ZT16-17). Black and white rectangles indicate the times of night and day, respectively. Time of SF indicated by vertical black dotted lines. (**G**,**H**) Alternation scores during baseline and SF conditions at four different zeitgeber times (ZT; *P < 0.01). ZT17 and ZT23 represent early and late night, respectively. Night and day are indicated by black and white rectangles in panels E,F. Sample sizes (n) for panels G,H: entrained (SF = 9; AL = 10), arrhythmic (SF = 12; AL = 9). All animals remained in a 16:8 light-dark cycle throughout the study.

Entrainment to a daily feeding schedule is characterized by intense bouts of locomotor activity that begin several hours before the scheduled meal^10, 11^. This food anticipatory activity (FAA) is controlled by the FEO and thus exhibits robust circadian properties^12^. In the present study, FAA gradually developed during SF in light-entrained hamsters and in those animals made circadian-arrhythmic by the DPS protocol (Fig. 2A–F). In light-entrained hamsters, the locomotor rhythm was driven by two activity peaks: one associated with FAA during the 5 h prior to the scheduled mealtime, and one during the normal time of nocturnal activity (Fig. 2A and E). In DPS-arrhythmic hamsters, the rhythm in locomotion consisted solely of FAA (Fig. 2B and F). Light-entrained hamsters exhibited FAA ratios and durations of elevated activity that were similar to those previously reported for wild-type rats or mice^13–15^ (Fig. 2C and E). By comparison, FAA ratios were 56% higher in DPS hamsters compared to light-entrained animals when data were averaged over the last 5 days of SF (two-way ANOVA; F_1,18_ = 5.85, P = 0.028; Fig. 2D and F). These data show that animals can anticipate a meal even when the LEO is nonfunctional, and that DPS-arrhythmic animals are more responsive to SF than animals with an intact LEO. This latter point is consistent with a large body of work reporting that: 1) the SCN pacemaker normally operates in the FEO circuit to prevent FAA, 2) SCN lesions can facilitate longer, more intense periods of FAA, and 3) FAA rhythms can override SCN signals that suppress daytime arousal^16–21^. After 3 weeks of SF, both groups of hamsters were returned to *ad libitum* food access for 3 weeks. FAA returned to baseline within 24 h after the SF regimen had concluded (Fig. 2C and D).

### Scheduled feeding improves spatial memory in DPS hamsters

When entrained to a 16:8 h light-dark (LD) cycle, Siberian hamsters alternate their exploration pattern in a T-maze in a daily rhythm with maximum alternation rates occurring from ZT11 to ZT23^22^. Between ZT3 and ZT11, they do not exhibit alternation scores significantly above chance^22^. Unlike light-entrained animals, DPS-arrhythmic hamsters do not alternate in the T-maze at any circadian phase of the 24 h day^22^. Thus, the lack of a functional LEO in these animals significantly impairs their spatial memory.

Before starting the food schedule, *ad libitum* fed hamsters (both light-entrained and DPS) were tested in the T-maze between ZT4-5. Consistent with our previous work^22^, we found that neither light-entrained nor DPS-arrhythmic hamsters were able to alternate at this time (i.e., alternation scores were not significantly above 50% chance performance; Fig. 2C and D, Day 5, right axis). However, when tested 3 weeks later on the final day of SF, each group displayed significant alternation at this time point (Fig. 2C and D, Day 26, right axis). Thus, a 3-week SF regimen at ZT5 enabled light-entrained animals to perform in the SA spatial memory test at a circadian phase when they usually cannot (ZT5), and rescued spatial memory in the DPS animals. We performed two control experiments to determine whether memory rescue in DPS hamsters was specific to the SF interval or to the LD cycle. Those experiments showed that T-maze performance by DPS hamsters remained entrained to the time of SF even when the LD cycle was shifted, or when the animals were placed in constant darkness (Supplementary Fig. 1). Acute arousal caused by overnight food deprivation did not improve alternation performance at ZT5 in light-entrained animals either (55.6 ± 5.2%, n = 10; P > 0.05). As such, memory rescue seems to result from entrainment to the SF period and not to the LEO.

### Scheduled feeding induces a daily rhythm in cognition in DPS hamsters

Following the 21 days of SF, all animals were returned to *ad libitum* feeding for 3 weeks. During this *ad libitum* period, hamsters were retested on the SA task several times at 2-day intervals (Fig. 1). We did this to test whether memory improvements were dependent on the continued presence of SF, and to show that memory improvements were not a byproduct of potential metabolic changes occurring during SF. Each animal was tested at four different time points of the 24 h cycle (ZT5, ZT11, ZT17, and ZT23 as illustrated in Fig. 1).

We first tested SA behavior in DPS-arrhythmic animals at ZT5 and they performed well (Fig. 2H). Two days later, we retested those animals at ZT17 and they failed to alternate (Fig. 2H). ARR hamsters were then retested at ZT5 to see if their poor performance was due to a waning effect of the SF on memory (Supplementary Fig. 2). It was not—DPS animals performed well during the second test at ZT5. In subsequent tests, DPS animals failed to alternate at ZT11, but performed well at ZT23 (Fig. 2H). Thus, memory rescue in the DPS hamsters was not an artifact of the circadian phase at which hamsters were tested (Supplementary Fig. 2). Taken together, the results show that a daily rhythm in spatial working memory was induced by SF even though locomotor activity remained arrhythmic, which suggests that these two functions are separable in the absence of a functional LEO.

Light-entrained hamsters continued to exhibit alternation behavior in the T-maze at ZT5 and at two other circadian phases when they normally perform well (ZT17 and ZT23; Fig. 2G). However, these light-entrained animals could no longer alternate better than chance at ZT11 (Fig. 2G). One possible explanation for this result is that the SF regimen phase-delayed the rhythm in SA behavior by several hours without phase-shifting the rhythm in locomotor activity, which remained nocturnal (Fig. 2A). Our interpretation of this finding is based on our prior work showing that light-entrained hamsters consistently failed at both the SA and novel object recognition tasks from ZT3-ZT7, but performed well from ZT11-ZT23^6, 22^. More time points would be needed, however, to confirm whether spatial memory rhythms in the present study were truly phase-shifted. These results complement observations made by others who found that scheduled normocaloric feeding at midday does not affect the phase of the locomotor activity rhythm in nocturnal rodents^23^, but is associated with c-Fos activation and phase-shifts of clock genes in “memory” circuits of the prefrontal cortex and hippocampus (i.e., circuits important for SA behavior)^24–26^.

Motivation to explore the maze did not change in either the light-entrained or DPS condition across the 24 h LD cycle or in response to SF as indicated by the number of arm entries in the T-maze (Supplementary Table 1). Positional bias scores different from 50% would suggest that the animals are perseverating on one side of the maze and that their arm choices are not being guided by spatial exploration. Under this circumstance, a spatial working memory deficit would not be genuine. Our data show that even when the light-entrained or DPS hamsters failed the SA test, they still showed a high number of arm entries (e.g., motivation to explore the environment) and no positional biases (Supplementary Table 1).

### Identifying brain regions activated by scheduled feeding in LEO-impaired animals: the suprachiasmatic nucleus

In an effort to identify some of the neural adaptations that accompanied memory improvement, we surveyed activity in specific brain regions thought to be involved with both food entrainment and declarative memory by quantifying c-Fos expression in those regions^6, 27, 28^. These regions included the SCN, the medial and lateral divisions of the septum, the hippocampal dentate gyrus, the dorsal, ventral, and compact zones of the hypothalamic dorsomedial nucleus (DMH), and the amygdala. Light-entrained and DPS-arrhythmic hamsters were sacrificed on the last day of SF between ZT4-5 to image neurons that were active in both groups during the height of the FAA response (Fig. 2E and F). *Ad libitum* fed animals were used as a control to examine the effects of food entrainment independent of LEO status. Neural excitability at ZT5 was measured by immunohistochemical detection of the immediate-early gene c-Fos protein. This regulatory transcription factor is anatomically distributed throughout the brain. It is rapidly increased from low basal levels by specific forms of patterned spike firing coincident with information processing, and has been employed over the past two decades as a histological record of circuit activity involved with different behavioral experiences, although it is not a perfect proxy of all neural activity^29, 30^.

**Figure 3:**
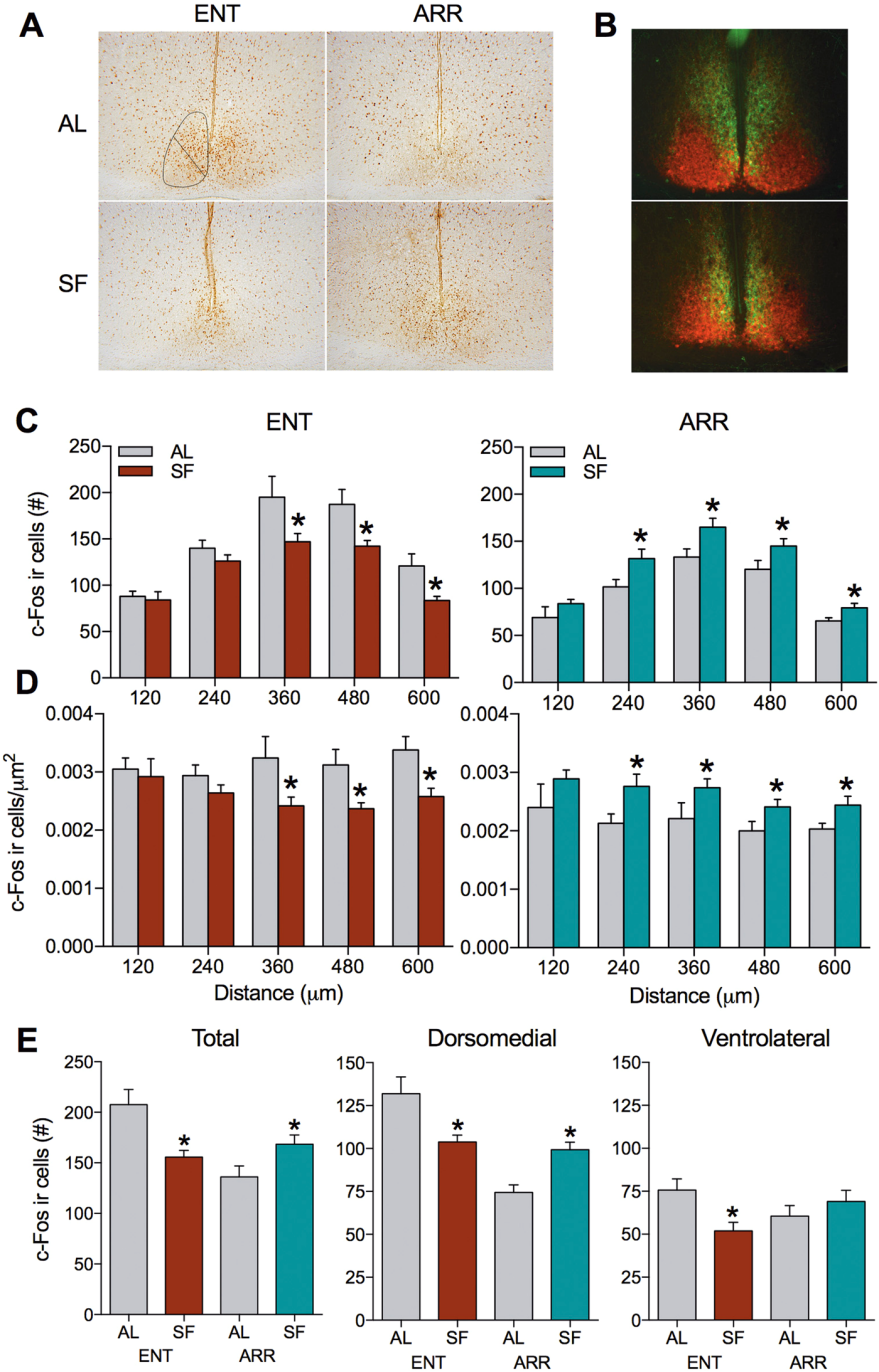
Induction of c-Fos expression in the SCN occurred primarily in the dorsomedial region along the mid to caudal SCN axis. (**A**) Representative tissue sections showing typical patterns of c-Fos expression in entrained (ENT) and in DPS-arrhythmic (ARR) animals that underwent either the scheduled feeding (SF) regimen or were fed *ad libitum* (AL). (**B**) Representative tissue sections from the mid (upper section) and caudal (lower section) SCN showing patterns of immunoexpression of vasopressin-neurophysin (green) and vasoactive intestinal polypeptide (red). These sections were used as templates for demarcating dorsomedial and ventrolateral SCN boundaries for the cell counts. (**C**) Number of cells counted at sequential levels of the SCN along its rostrocaudal axis. Sections were taken at intervals of 120 μm (* indicates significant differences between AL and SF conditions; P < 0.05, n = 6 per group). (**D**) Data from C given as density of c-Fos IR cells. (**E**) Number of c-Fos IR cells in the dorsomedial and ventrolateral subregions of the SCN, as well as total cell counts (* indicates P < 0.05 compared to AL condition).

As previously demonstrated for other rodents^16, 18, 19^, SF reduced c-Fos expression in the SCN of light-entrained hamsters compared to animals fed *ad libitum*. This reduction was localized to the mid-caudal SCN (Fig. 3A–D, left panels), but was also seen in both the dorsomedial and ventrolateral regions of the nucleus (Fig. 3E). By contrast, SF increased c-Fos immunoreactivity (IR) in the SCN of DPS hamsters compared to DPS animals fed *ad libitum*. In DPS animals, activation of the SCN was observed in the dorsomedial region only and was greatest in the mid to caudal SCN (Fig. 3A–D, right panels). Under the a*d libitum* condition, light-entrained hamsters had 25–30% more c-Fos expression in the SCN than did DPS hamsters.

### Identifying brain regions activated by scheduled feeding in LEO-impaired animals: the septum and hippocampus

**Figure 4:**
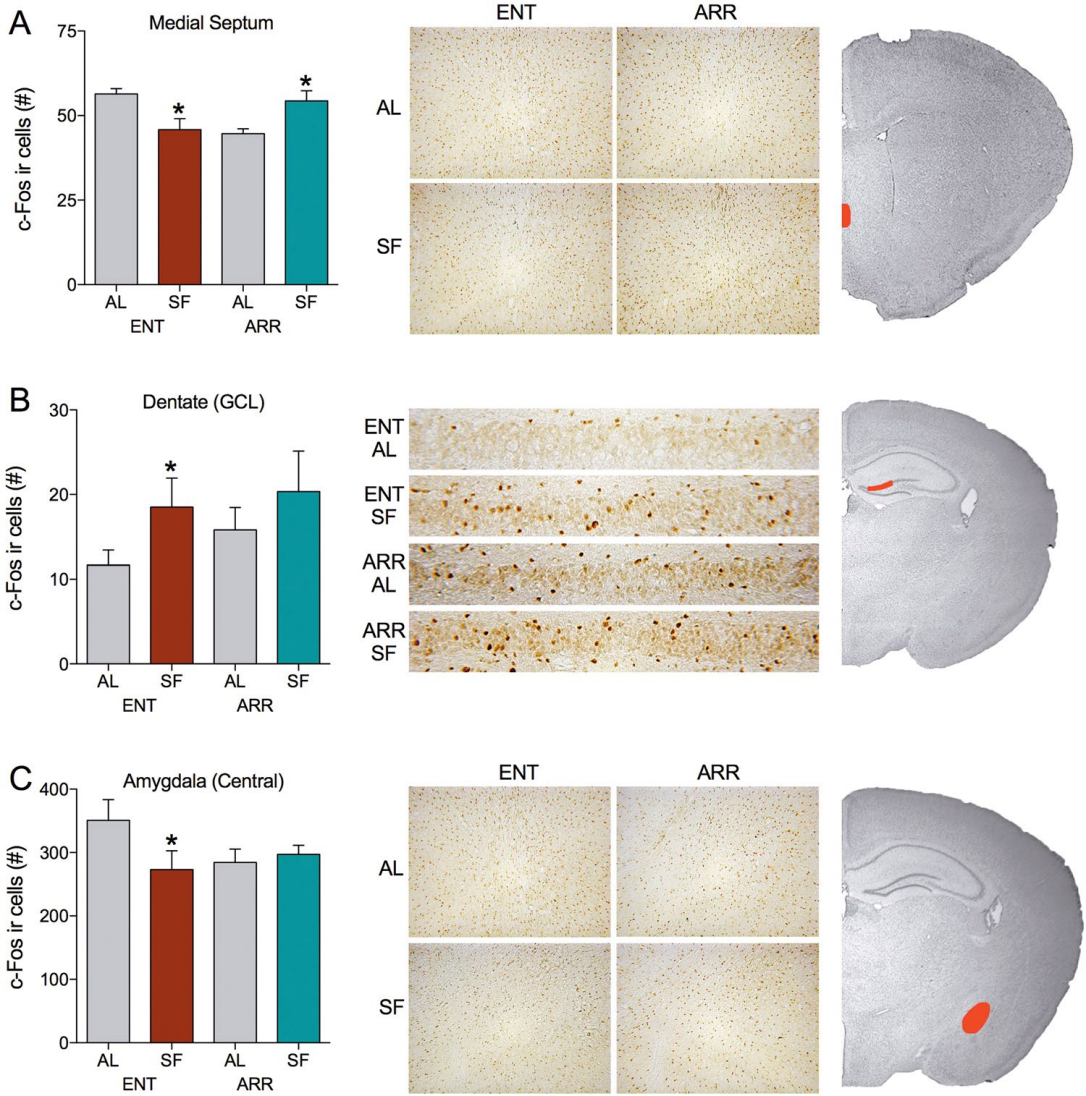
SF equalized c-Fos expression among ENT and ARR animals in specific non-SCN brain regions. Bar graphs show mean (±SE) number of c-Fos IR cells in each group for the (**A**) medial septum, (**B**) granule cell layer of the dentate gyrus, and (**C**) central nucleus of the amygdala (*P < 0.05 compared to AL condition). The middle column of panels shows representative c-Fos labeling in brain sections from all four groups of animals. Red areas in hamster coronal sections on the right column indicate the areas in which cells were counted.

**Figure 5:**
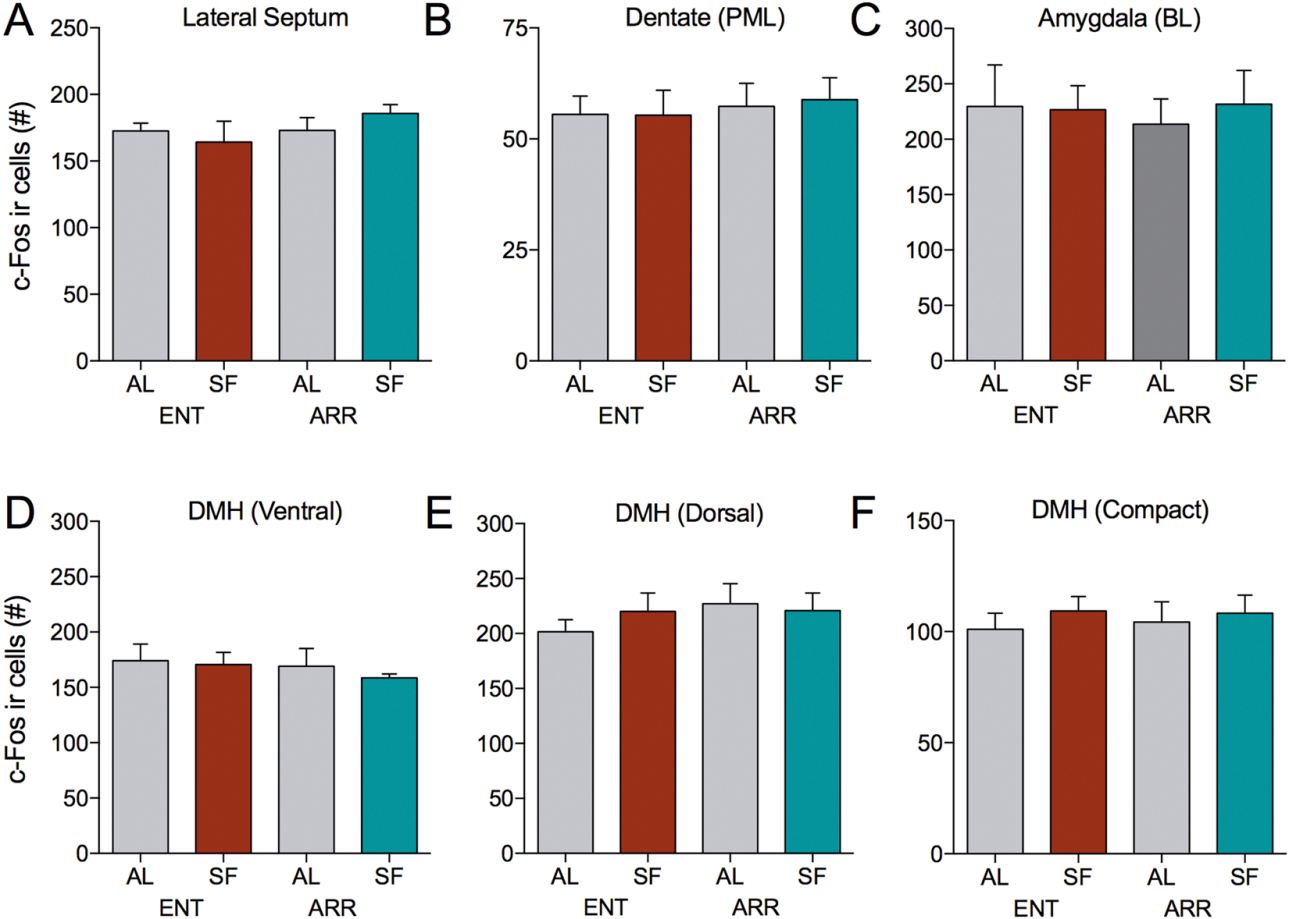
SF had no effect on c-Fos expression in these brain regions. (**A**) lateral septum, (**B**) polymorphic layer of the dentate gyrus, (**C**) basolateral nucleus of the amygdala, (**D**) ventral region of the dorsomedial hypothalamic nucleus (DMH), or (**E**) dorsal region of the DMH, and (**F**) compact nucleus of the DMH.

In the septal complex (Figs 4 and 5), we found no c-Fos changes in the lateral division of the septum. Light-entrained- and DPS-animals did not differ in lateral septum c-Fos IR and this lack of difference was not influenced by SF (Fig. 5A). As observed in the SCN, light-entrained hamsters exhibited more c-Fos positive cells in the medial septum than DPS-arrhythmic hamsters. However, SF equalized activity across the light-entrained and DPS groups by lowering expression of c-Fos in the light-entrained medial septum, and raising it in the DPS medial septum (Fig. 4A). Thus, SF induced changes in c-Fos activity in the medial septum that paralleled those in the SCN. Both brain regions displayed less activation in the DPS *ad libitum* state and both were stimulated by SF (Figs 3 and 4).

Consistent with other activity mapping surveys in food-entrained rats and mice^27, 31^, c-Fos IR increased significantly in the granule layer, but not the polymorphic layer, of the dentate gyrus in light-entrained hamsters (Figs 4B and 5B). On the other hand, SF had no statistically significant effect on the number of c-Fos positive granule cells in DPS hamsters. This lack of effect in DPS hamsters might be due to the fact that these animals already had higher levels of c-Fos activation under *ad libitum* conditions than did light-entrained animals (Fig. 4B). It is possible that SF could not increase this activation much further. Collectively, the c-Fos data suggest that SF alters neural activity in the dorsomedial SCN, medial septum, and perhaps the hippocampus, and does so at the same times of day when SF induces arousal.

Several studies have implicated the DMH as part of a neural circuit that defines the FEO^32, 33^. Other studies have found that the DMH is dispensable for FAA behavior^34–36^. Elevated expression levels of c-Fos have been noted in the DMH during feeding, but not during the period specific to FAA^37^. Our animals were sacrificed in the hour prior to the onset of feeding (Fig. 1). Here, SF had no effect on any subregion in the DMH in either light-entrained or DPS hamsters (Fig. 5D–F). Our results are more consistent with the interpretation that the DMH plays a role in signaling satiety rather than signaling the expectation of food^37^.

In addition to the structures discussed here, we evaluated c-Fos expression in the amygdala because increases in c-Fos expression have been previously reported in the central nucleus (CEA) and basolateral amygdala (BLA) of rats submitted to SF or to schedules of palatable food access^31^. SF reduced CEA activation during the FAA window in light-entrained hamsters relative to controls fed *ad libitum*. However, c-Fos IR in the CEA remained unaffected by food availability in DPS animals (Fig. 4C). Neither light-entrained or DPS animals exhibited differences in IR for c-Fos in the BLA (Fig. 5C), nor was c-Fos expression in the BLA affected by SF.

## Discussion

The DPS protocol eliminates circadian timing within the SCN without the need for genetic modifications or surgical or pharmacological interventions to achieve chronic arrhythmia^8^. Persistent circadian arrhythmia is associated with substantial impairments in spatial and recognition memory^6, 22^. The present study used a scheduled feeding paradigm to impose a daily rhythm in behavior in an attempt to improve memory performance. Spatial working memory was rescued by this treatment and persisted for at least 3 weeks after cessation of SF in arrhythmic hamsters. Our findings imply that the DPS protocol impairs the LEO, but leaves the FEO intact, a finding that will be useful for future experiments aimed at isolating FEO function. Perhaps the most provocative finding here is that the FEO had a demonstrable role in declarative memory that persisted for several weeks after SF and during *ad libitum* feeding conditions. Typically, for light-entrained animals that have resumed normal feeding for one or more weeks after SF, the persistence of coupling between FEO and behavior can only be revealed by several days of fasting^12^. By contrast, DPS animals did not have to be food-deprived to show phase-specific persistence of memory performance during the previous mealtime window even after several weeks of unlimited food access. Memory improvement was not accompanied by any behavioral activities that could be interpreted as FAA. It is not immediately clear how the FEO can support declarative memory processing, but the effect might be explained in part by the extent of the circuitry that is mobilized during SF.

The FEO represents a distributed neural network that supports FAA and corollary activities^38^. For these functions, the FEO is not reliant on any one structure within the network, but employs redundant circuitry during the premeal activity window that drives attention^27, 31, 39–42^, affect or motivation^43–45^, and oscillatory states important for information processing such as hippocampal theta^28, 46^. At the crossroads of this circuitry is the SCN. When the SCN assimilates and computes light information, its competing role as a component in the FEO circuitry is to gate and shape the FAA window. Under normal circumstances, its general silence serves to facilitate activity in other parts of the FEO network. The data presented here suggest that a different dynamic is at play when the LEO’s operation has been impaired.

LEO impairment in SCN-intact Siberian hamsters results in memory deficits, ostensibly from altered signaling between the SCN and downstream targets in the septohippocampus^6, 47^. Our results suggest that SF can compensate for deficient SCN signaling at times of day that are coincident with a meal and suggest that the FEO’s organizing effects on cognition versus locomotor activity can be teased apart when LEO function is absent. This supposition is supported by experiments in which rats that were made circadian-arrhythmic by constant light recovered SCN rhythms in clock gene expression after daily SF^48, 49^. Why FEO stimulation of DPS hamsters should result in increased dorsomedial activation of the SCN and not general activity suppression of the nucleus as seen in light-entrained hamsters is an important future question.

The long-term effects of SF on cognition might be explained by neural adaptations in basal forebrain, prefrontal cortex, and hippocampus that prime acetylcholine release during the FAA window^39–41^. For example, previous work suggests that hippocampal theta rhythms, activated in part through septal cholinergic fibers^50^, are entrained by food availability and that circadian information about food availability is encoded within the hippocampus^46^. These adaptations might be especially long-lived in arrhythmic hamsters because the most salient—and arguably one of the only—zeitgebers available to them is food availability. DPS hamsters may be more responsive to food as a time cue, and may retain temporal information about previously reliable food cues even after returning to *ad libitum* food consumption, when they are unable to use the light-dark cycle as a timing cue.

A role for the FEO in support of declarative memory makes most sense if one considers the cognitive demands required to find food in the wild. Animals often consume food only after completing a prolonged period of foraging or hunting, activities that demand a significant investment of cognitive resources linked to: planning, decision making, improvisation, spatial navigation, perceptual learning, and associative memory^51–56^. These foraging periods—or food anticipation windows—are timed to a specific ecological niche that allows foragers to avoid predation or hunters to find prey when they are most vulnerable. Built into the biology of most animals, therefore, is the ability to mobilize a vast array of cognitive skills at some recurring time each day that will improve their chances of finding food.

Given its evolutionary advantage for fitness and survival, humans might also have conserved the ability to set and shift optimal brain function to predictable windows of food availability, but not when food is generally available. This phenomenon is likely reliant on stimulating the FEO with repetitively spaced food cues^57^. People that cannot entrain optimally to the light-dark schedule are at heightened risk for age- and disease-related cognitive impairment^2, 3, 5^. Our findings are the first to show the treatment potential of stimulating a secondary circadian oscillator that can entrain to 24-h food cycles. This secondary oscillator, the FEO, might have more latitude to improve memory performance when the LEO has been compromised. Because FEO function is maintained through advanced age^58, 59^, it might represent a valuable therapeutic target to slow progression of Alzheimer’s disease in cases where LEO impairment is suspected to accelerate cognitive decline.

## Experimental procedures

### Subjects and housing conditions

Siberian hamsters (*Phodopus sungorus*) were bred in the laboratory in a 16:8-h light-dark (LD) cycle (lights on at 0200 h, PST) at an ambient temperature of 22 °C. Animals were provided with cotton batting for nesting material. Water was available *ad libitum*. Food (Purina chow #5015) was available *ad libitum* prior to, and then after the period of scheduled feeding. All experimental procedures were approved by Stanford University’s Administrative Panel on Laboratory Animal Care (Animal Use Protocol Number: 14988) and were conducted in accordance with the *NIH Guide for the Care and Use of Laboratory Animals*. Male and female hamsters were used in all experiments.

Housing and lighting conditions were as described previously^22^. Locomotor activity was measured by passive infrared motion detectors mounted directly above the tip of the water bottle sipper tube. Mounted in this manner, the detectors were able to detect drinking behavior and most movement within the cage. Activity bouts were summed in 10-min intervals and stored on computer. The times of day when spatial memory was tested are given by zeitgeber time (ZT) where ZT0 = time of lights-on and ZT16 = time of lights-off in the animal rooms.

### The disruptive phase shift (DPS) protocol

Hamsters were rendered circadian-arrhythmic by the DPS protocol at 2–4 months of age^22^. The animals were separated and singly housed in activity recording chambers programmed to the same photoperiod as the colony room (LD 16:8, lights-on at 0200 PST). Fourteen days later, lights in the chambers were turned on for 2 h beginning 5 h after lights-off (i.e., a 2-h light pulse from ZT21-23). On the next day, the LD cycle was phase delayed by 3 h so that dark onset occurred 3 h later than on the previous night (lights-on at 0500 PST). Animals remained in the 16:8 LD cycle thereafter and locomotor activity was monitored continuously.

### Scheduled feeding protocol

Scheduled feeding was initiated by completely depriving the animals of food for 13 h beginning at the time of lights-off in the colony room or recording chambers and continuing deprivation until ZT5 the next day. This ensured that the animals were hungry once food was presented at ZT5, where they were allowed to consume as much as they wanted for the next 8 h (i.e., ZT5-13). From here, the time window when food was available was gradually shortened by 30 min each day by advancing the end of the feeding period until it was reduced to 4 h (i.e., over the course of 8 days). During the first 9 days of scheduled feeding, animals were weighed every other day to ensure that they did not temporarily lose more than 20% of their baseline body mass. For daily feeding at ZT5, food (>100 g) was provided in the overhead hopper. Once the feeding window had elapsed, all remaining food from the hopper or hoarded in the bedding was removed from the cage. After the first 9 days of scheduled feeding, body mass stabilized and food intake increased. The animals were able to consume their baseline daily intake of food within the 4-h period.

### Animal handling

Hamsters were gently handled daily for 5 min over 7–10 days prior to baseline testing using a procedure that gradually introduced them to the experimenter. During the first 3 days, the experimenter’s hand was placed in the cage and the hamster was allowed to sniff and crawl over the hand. On days 4 and 5, each hamster was held in the experimenter’s hand without removing the animal from the cage. Over the remaining days, the hamsters were removed from the cage, held in both hands, and allowed to crawl along the experimenter’s arm. The animals were considered ready for testing when they could be picked up without retreating from the experimenter and exhibited no obvious signs of stress (e.g., vocalizations, escape behavior, rapid respiration). Once memory testing began, animals were no longer handled.

### Spontaneous alternation in the T-maze

SA is based on the natural tendency of rodents to consecutively alternate between left and right arm choices during exploration of a T-maze. Because animals are intrinsically motivated to explore new environments, SA behavior does not require a food reward or any training sessions to elicit alternation behavior (see Supplementary Text for further details). A sliding door separated the start chamber from the rest of the apparatus, comprised of an alleyway that led to a choice point at the intersection of the stem with the left and right arms of the maze. A divider panel was centered at the intersection of the “T” and extended a short distance into the stem arm. Dimensions of the T-maze and lighting conditions were as described^22^.

To test an animal for SA behavior, an animal is confined to the start chamber located at the end of the stem arm of a T-maze for 60 s. The door is then raised and the animal is free to explore the entire maze for 7 min. In a typical SA test, the animal moves along the stem and then enters the left or right arm of the maze and then returns to, or close to, the start area. Afterwards, it typically travels back along the stem arm and then enters either the lateral arm that it chose on its prior visit, or enters the opposite arm. An alternation attempt was scored when all four feet of the hamster entered one of the lateral arms, re–entered the stem, and then entered the lateral arm opposite the one previously chosen. Re-entry into the same arm was a non-alternation. Performance was operationally defined by the percentage of time a hamster alternated upon arriving at the divider panel (i.e., the number of alternations observed/the number of alternation attempts ×100). The T–maze was cleaned with 70% ethanol, dried, and ventilated for a few minutes between trials. Alternations were scored by observers during the task.

### Immunocytochemistry

Hamsters were euthanized with an overdose of isoflurane gas and perfused transcardially with 30cc of 0.01 M phosphate buffered saline (PBS, pH 7.4) followed by 30cc of 4% paraformaldehyde in 0.01 M PBS (PFA, pH 7.4). Brains were then removed, post-fixed overnight in PFA, and cryoprotected using 20% sucrose in PBS for 24 h. Tissue was then sectioned at 30 µm on a cryostat into PBS. Sections were transferred to PBS antifreeze solution and stored at −20 °C until staining by immunocytochemistry.

To visualize cells containing c-Fos protein, tissue sections were washed in PBS 5 × 5 min and transferred to 0.3% H_2_0_2_ for 30 min. This was followed by: 3 × 5 min rinses in PBS with 0.3% Triton X-100 (PBSX, Sigma); blocking in 10% normal donkey serum (NDS, Jackson ImmunoResearch Laboratories; cat. no. 017 000 121) and 20% Avidin (Vector Laboratories; cat. no. SP-2001) in PBSX for 1 h; and incubation overnight in primary rabbit anti-FOS antibody (1:10 000, Santa Cruz Biotechnology) with 3% NDS and 20% biotin in PBSX. The following morning, the sections were rinsed with PBSX 3 × 5 min and incubated in secondary biotinylated donkey anti-rabbit antibody (1:250, Jackson ImmunoResearch Laboratories) and 3% NDS in PBSX for 1 h. The tissue was then rinsed 3 × 5 min in PBSX before being incubated with an avidin biotin horseradish peroxidase kit (1:200, ABC; standard Vectastain elite kit, Vector Laboratories, cat. no. PK- 6100) for 1 hr. The signal was developed by incubation in diaminobenzidine (DAB) activated using H_2_0_2_ for 90 sec.

For dual-label immunofluorescence, tissue sections were blocked with Tris-buffered saline (TBS) containing 10% normal goat serum (Vector Laboratories) and 0.4% Triton X-100, then incubated overnight at 4 °C in a primary antibody cocktail (anti-vasopressin-neurophysin: 1:500, mouse, PS 45; a generous gift from Dr. Harold Gainer^60^; anti-vasoactive intestinal peptide, 1:10000, rabbit, Immunostar, cat no 20077). The following day, tissue was rinsed in TBS with 0.4% Triton X-100 and 2% normal goat serum (TTG) 5 × 5 min and incubated in a secondary antibody cocktail that consisted of Alexa Fluor 488 goat anti-mouse and Alexa Fluor 594 goat anti-rabbit antibodies (Molecular Probes) in TTG for 2 h. After final rinses in TTG (5 × 5 min), sections were mounted on slides and coverslipped with Prolong Mounting Medium (Molecular Probes).

### Cell counting

Tissue sections stained with c-Fos antibody were mounted on Superfrost^TM^ Plus slides, coverslipped, and examined under a light microscope interfaced to a computer workstation. Digital images of coronal sections containing the following brain areas were captured by a Qimaging QIClick CCD camera: the suprachiasmatic nucleus (**SCN**), the ventral portion of the lateral septum (**VLS**), the medial septum (**MS**), the granule layer of the dentate gyrus (**DGG**), the polymorphic layer of the dentate gyrus (**DGP**), the dorsomedial hypothalamus (**DMH**), and the basolateral (**BLA**) and central (**CEA**) amygdala. All c-Fos labeled nuclei with staining intensities that were ≥2x background were quantified manually at 200x magnification using the Cell Counter plugin in ImageJ 1.48 v (National Institutes of Health). Regions of interest (ROI) were created for each structure by referencing the stereotaxic atlas of Paxinos and Franklin, *The Mouse Brain in Stereotaxic Coordinates* (3rd edition), and cross-referencing these selections with nissl-stained tissue sections prepared from the Siberian hamster brain. Composite photos were stitched together using Adobe Photoshop if the ROI was too large to fit into one field of view, as was the case for analyses of the DMH, BLA, and CEA.

Unilateral cell counts were performed in the dorsomedial shell and ventrolateral core of the SCN, as well as in the VLS, DGG, DGP, and dorsal/ventral DMH. The MS, BLA, and CEA were quantified bilaterally. For the SCN, the number of c-Fos immunoreactive cells was tabulated from two successive tissue sections prepared from each hamster (n = 6 animals per entrainment/feeding condition; 24 total). For analysis of the VLS, MS, DMH, DGG, and DGP, one representative section was chosen per animal at the same anterior-posterior position. There were variations between hamsters in the curvature of the DGG. To ensure that the ROI selection would fit every tissue section appropriately, the DGG was straightened using the “straighten” function in ImageJ. A single examiner (N.F.), who was blind to the experimental group to which each animal was assigned, performed all the cell counts included in this study.

### Data analysis

Performance on the SA test was determined by a one-sample t-test to determine whether scores were statistically different from random chance performance (i.e., alternation = 50%). A score of positional bias was created to check for left-right biases in the T-maze arms. Positional bias was calculated as: time on the right/(time on the left + time on the right) × 100, so that a score that is significantly <50% indicates a left bias, and >50% indicates a right bias. Changes in the number of arm entries and in positional bias were evaluated by one-way analysis of variance (ANOVA) with repeated measures. Dunnett’s correction was set at P = 0.05 and used for all subsequent pairwise comparisons with the baseline condition serving as the control group. Data are presented as mean ± SEM. Siberian hamsters do not exhibit any sex differences in SA performance, arm entries, or positional bias^22^, so data from males and females were combined. Most groups were balanced by sex, but in some cases this was not possible (i.e., groups with odd number sample sizes).

FAA ratios were calculated in the same manner for DPS and entrained animals. Visual inspection of the actograms of DPS hamsters indicated that these animals exhibit a high level of FAA beginning at the time of lights-on that persists until food is made available. Therefore, the total amount of activity during the 5 h prior to feeding (ZT0-5) was divided by the total amount of activity for the daily cycle (ZT0-24). For the waveforms, total hourly amounts of locomotor activity were averaged for all animals in each group and plotted across 24 h. Differences in the number of c-Fos cells between AL and SF groups were tested statistically by t-tests.

## Electronic supplementary material

Supplementary Information

## References

[1] Weinert D (2000). Age-dependent changes of the circadian system. Chronobiology International.

[2] Tranah GJ (2011). Circadian activity rhythms and risk of incident dementia and mild cognitive impairment in older women. Annals of Neurology.

[3] Walsh CM (2014). Weaker circadian activity rhythms are associated with poorer executive function in older women. Sleep.

[4] Kent BA (2014). Synchronizing an aging brain: can entraining circadian clocks by food slow Alzheimer’s disease?. Frontiers in Aging Neuroscience.

[5] Landry GJ, Liu-Ambrose T (2014). Buying time: a rationale for examining the use of circadian rhythm and sleep interventions to delay progression of mild cognitive impairment to Alzheimer’s disease. Frontiers in Aging Neuroscience.

[6] Ruby NF (2008). Hippocampal-dependent learning requires a functional circadian system. Proceedings of the National Academy of Sciences USA.

[7] Larkin JE, Yokogawa T, Heller HC, Franken P, Ruby NF (2004). Homeostatic regulation of sleep in arrhythmic Siberian hamsters. American Journal of Physiology - Regulatory, Integrative and Comparative Physiology.

[8] Grone BP (2011). Acute light exposure suppresses circadian rhythms in clock gene expression. Journal of Biological Rhythms.

[9] Pellman BA (2015). Time-specific fear acts as a non-photic entraining stimulus of circadian rhythms in rats. Scientific Reports.

[10] Boulos Z, Rosenwasser AM, Terman M (1980). Feeding schedules and the circadian organization of behavior in the rat. Behavioural Brain Research.

[11] Richter CP (1922). A behavioristic study of the rat. Comparative Psychology Monographs.

[12] Mistlberger RE (1994). Circadian food-anticipatory activity: formal models and physiological mechanisms. Neuroscience & Biobehavioral Reviews.

[13] Landry GJ, Yamakawa GR, Mistlberger RE (2007). Robust food anticipatory circadian rhythms in rats with complete ablation of the thalamic paraventricular nucleus. Brain Research.

[14] Landry GJ (2011). Evidence for time-of-day dependent effect of neurotoxic dorsomedial hypothalamic lesions on food anticipatory circadian rhythms in rats. PLoS One.

[15] Storch KF, Weitz CJ (2009). Daily rhythms of food-anticipatory behavioral activity do not require the known circadian clock. Proceedings of the National Academy of Sciences USA.

[16] Acosta-Galvan G (2011). Interaction between hypothalamic dorsomedial nucleus and the suprachiasmatic nucleus determines intensity of food anticipatory behavior. Proceedings of the National Academy of Sciences USA.

[17] Angeles-Castellanos M, Salgado-Delgado R, Rodriguez K, Buijs RM, Escobar C (2010). The suprachiasmatic nucleus participates in food entrainment: a lesion study. Neuroscience.

[18] Escobar C (2007). Unpredictable feeding schedules unmask a system for daily resetting of behavioural and metabolic food entrainment. European Journal of Neuroscience.

[19] Challet E, Jacob N, Vuillez P, Pévet P, Malan A (1997). Fos-like immunoreactivity in the circadian timing system of calorie-restricted rats fed at dawn: daily rhythms and light pulse-induced changes. Brain Research.

[20] Holloway WR, Tsui HW, Grota LJ, Brown GM (1979). Melatonin and corticosterone regulation: feeding time or the light:dark cycle?. Life Sciences.

[21] Feillet CA, Mendoza J, Pevet P, Challet E (2008). Restricted feeding restores rhythmicity in the pineal gland of arrhythmic suprachiasmatic-lesioned rats. European Journal of Neuroscience.

[22] Ruby NF (2013). Spatial memory and long-term object recognition are impaired by circadian arrhythmia and restored by the GABAA antagonist pentylenetetrazole. PLoS One.

[23] Mendoza J, Graff C, Dardente H, Pevet P, Challet E (2005). Feeding cues alter clock gene oscillations and photic responses in the suprachiasmatic nuclei of mice exposed to a light/dark cycle. Journal of Neuroscience.

[24] Angeles-Castellanos M, Mendoza J, Escobar C (2007). Restricted feeding schedules phase shift daily rhythms of c-Fos and protein Per1 immunoreactivity in corticolimbic regions in rats. Neuroscience.

[25] Wakamatsu H (2001). Restricted-feeding-induced anticipatory activity rhythm is associated with a phase-shift of the expression of mPer1 and mPer2 mRNA in the cerebral cortex and hippocampus but not in the suprachiasmatic nucleus of mice. European Journal of Neuroscience.

[26] Waddington Lamont E (2007). Restricted access to food, but not sucrose, saccharine, or salt, synchronizes the expression of Period2 protein in the limbic forebrain. Neuroscience.

[27] Blum ID, Lamont EW, Rodrigues T, Abizaid A (2012). Isolating neural correlates of the pacemaker for food anticipation. PLoS One.

[28] Poulin AM, Timofeeva E (2008). The dynamics of neuronal activation during food anticipation and feeding in the brain of food-entrained rats. Brain Research.

[29] Morgan JI, Curran T (1986). Role of ion flux in the control of c-Fos expression. Nature.

[30] Sagar SM, Sharp FR, Curran T (1988). Expression of c-Fos protein in brain: metabolic mapping at the cellular level. Science.

[31] Verwey M, Khoja Z, Stewart J, Amir S (2007). Differential regulation of the expression of Period2 protein in the limbic forebrain and dorsomedial hypothalamus by daily limited access to highly palatable food in food-deprived and free-fed rats. Neuroscience.

[32] Gooley JJ, Schomer A, Saper CB (2006). The dorsomedial hypothalamic nucleus is critical for the expression of food-entrainable circadian rhythms. Nature Neuroscience.

[33] Landry GJ (2011). Evidence for time-of-day dependent effect of neurotoxic dorsomedial hypothalamic lesions on food anticipatory circadian rhythms in rats. PLoS ONE.

[34] Landry GJ, Simon MM, Webb IC, Mistlberger RE (2006). Persistence of a behavioral food-anticipatory circadian rhythm following dorsomedial hypothalamic ablation in rats. American Journal of Physiology - Regulatory, Integrative and Comparative Physiology.

[35] Landry GJ, Yamakawa GR, Webb IC, Mear RJ, Mistlberger RE (2007). The dorsomedial hypothalamic nucleus is not necessary for the expression of circadian food-anticipatory activity in rats. Journal of Biological Rhythms..

[36] Moriya T (2009). The dorsomedial hypothalamic nucleus is not necessary for food-anticipatory circadian rhythms of behavior, temperature or clock gene expression in mice. European Journal of Neuroscience.

[37] Gallardo CM (2014). Behavioral and neural correlates of acute and scheduled hunger in C57BL/6 mice. PLoS One.

[38] Davidson AJ (2009). Lesion studies targeting food-anticipatory activity. European Journal of Neuroscience.

[39] Ghiani CA (1998). Antagonism by abecarnil of enhanced acetylcholine release in the rat brain during anticipation but not consumption of food. Pharmacology Biochemistry & Behavior.

[40] Inglis FM, Day JC, Fibiger HC (1994). Enhanced acetylcholine release in hippocampus and cortex during the anticipation and consumption of a palatable meal. Neuroscience.

[41] Ismail N, Robinson GE, Fahrbach SE (2006). Stimulation of muscarinic receptors mimics experience-dependent plasticity in the honey bee brain. Proceedings of the National Academy of Sciences USA.

[42] Aston-Jones G, Chen S, Zhu Y, Oshinsky ML (2001). A neural circuit for circadian regulation of arousal. Nature Neuroscience.

[43] Mendoza J, Angeles-Castellanos M, Escobar C (2005). Differential role of the accumbens shell and core subterritories in food-entrained rhythms of rats. Behavioural Brain Research.

[44] Mendoza J, Angeles-Castellanos M, Escobar C (2005). Entrainment by a palatable meal induces food-anticipatory activity and c-Fos expression in reward-related areas of the brain. Neuroscience.

[45] Takase LF, Nogueira MI (2008). Patterns of fos activation in rat raphe nuclei during feeding behavior. Brain Research.

[46] Munn RG, Tyree SM, McNaughton N, Bilkey DK (2015). The frequency of hippocampal theta rhythm is modulated on a circadian period and is entrained by food availability. Frontiers in Behavioral Neuroscience.

[47] Fernandez F (2014). Dysrhythmia in the suprachiasmatic nucleus inhibits memory processing. Science.

[48] Lamont EW, Diaz LR, Barry-Shaw J, Stewart J, Amir S (2005). Daily restricted feeding rescues a rhythm of period2 expression in the arrhythmic suprachiasmatic nucleus. Neuroscience.

[49] Nováková M, Polidarová L, Sládek M, Sumová A (2011). Restricted feeding regime affects clock gene expression profiles in the suprachiasmatic nucleus of rats exposed to constant light. Neuroscience.

[50] Fischer Y, Gähwiler BH, Thompson SM (1999). Activation of intrinsic hippocampal theta oscillations by acetylcholine in rat septo-hippocampal cocultures. Journal of Physiology.

[51] Collett M, Chittka L, Collett TS (2013). Spatial memory in insect navigation. Current Biology.

[52] Zhang S, Si A, Pahl M (2012). Visually guided decision making in foraging honeybees. Frontiers in Neuroscience.

[53] Griffin AS, Guez D (2014). Innovation and problem solving: a review of common mechanisms. Behavioural Processes.

[54] O’Donnell S, Logan CJ, Clayton NS (2012). Specializations of birds that attend army ant raids: an ecological approach to cognitive and behavioral studies. Behavioural Processes.

[55] Clarin TM, Ruczyński I, Page RA, Siemers BM (2013). Foraging ecology predicts learning performance in insectivorous bats. PLoS One.

[56] Schuster S, Wöhl S, Griebsch M, Klostermeier I (2006). Animal cognition: how archer fish learn to down rapidly moving targets. Current Biology.

[57] Sulzman FM, Fuller CA, Moore-Ede MC (1977). Feeding time synchronizes primate circadian rhythms. Physiology & Behavior.

[58] Walcott EC, Tate BA (1996). Entrainment of aged, dysrhythmic rats to a restricted feeding schedule. Physiology & Behavior.

[59] Mistlberger RE, Houpt TA, Moore-Ede MC (1990). Effects of aging on food-entrained circadian rhythms in the rat. Neurobiology of Aging.

[60] Ben-Barak YA, Russell JT, Whitnall MH, Ozato KE, Gainer HA (1985). Neurophysin in the hypothalamo-neurohypophysial system. I. Production and characterization of monoclonal antibodies. Journal of Neuroscience.

