# Supplementary material for: Scheduled feeding restores memory and modulates c-Fos expression in the suprachiasmatic nucleus and septohippocampal complex
Source: Sci Rep. 2017 Jul 28;7:6755. doi: 10.1038/s41598-017-06963-w (PMC5533780; doi:10.1038/s41598-017-06963-w)
Supplement: Supplementary file 1 — Supplementary Information [file 41598_2017_6963_MOESM1_ESM.doc]

**Supplemental Materials for:**

Scheduled Feeding Restores Memory and Modulates c-Fos Expression in the Suprachiasmatic Nucleus and Septohippocampal Complex

Norman F. Ruby^1*^, Nathan Fisher^1^, Danica F. Patton^1^, Matthew J. Paul^2^, Fabian Fernandez^3,4^, and H. Craig Heller^1,4^

^1^Biology Department, Stanford University, Stanford, CA 94305

^2^Department of Psychology, University at Buffalo, SUNY, Buffalo, NY 14260

^3^Departments of Psychology and Neurology, BIO5 Institute, and The Evelyn F. McKnight Brain Institute, University of Arizona, Tucson, AZ 85724

**
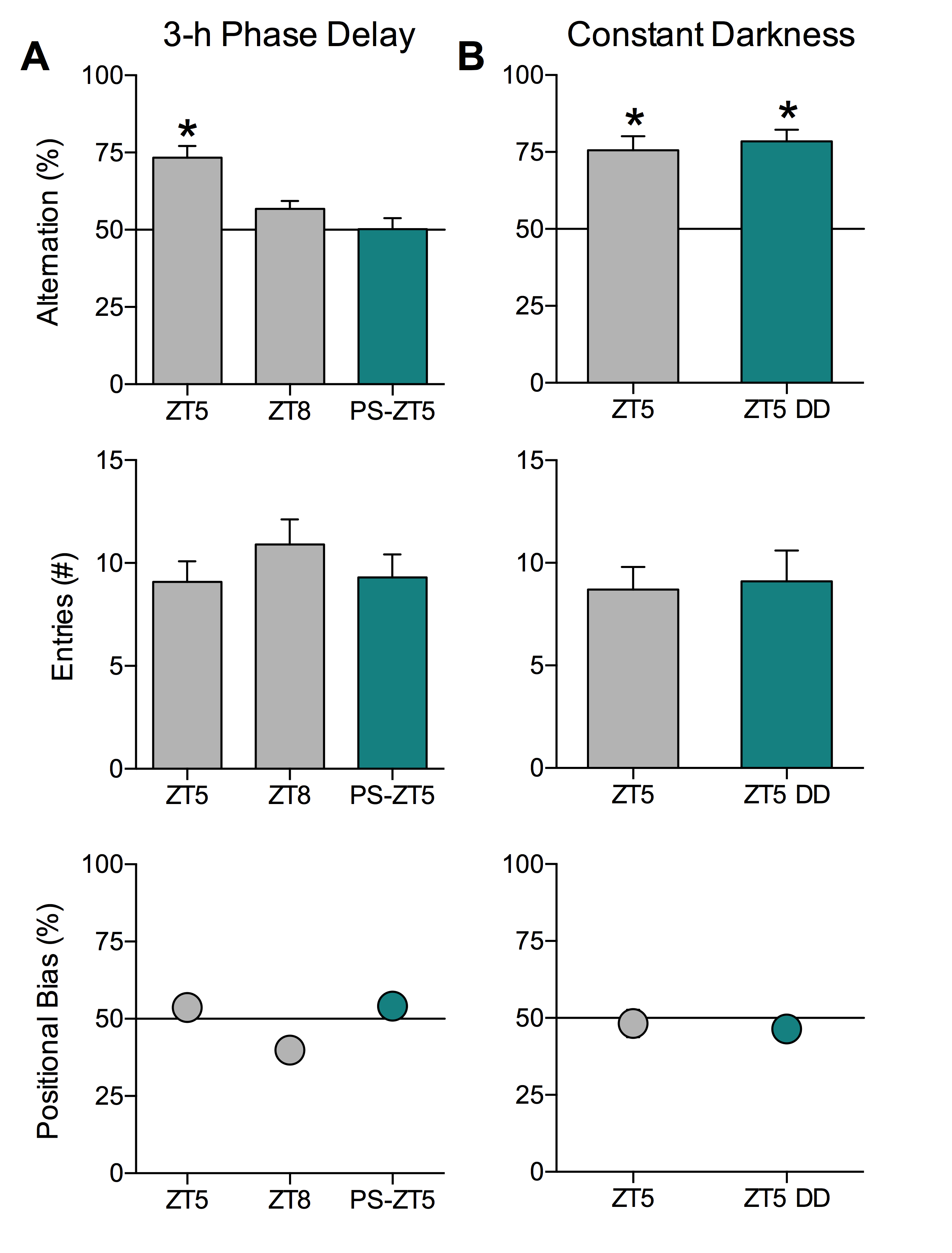
**

**Supplementary Figure 1. DPS-arrhythmic hamsters entrain their performance on the SA test to the feeding schedule, but not to the light-dark cycle.** (A, top panel) A separate group of DPS hamsters from those used in the main experiments were assessed for spontaneous alternation 14 days after SF ended (n=10). These animals performed well at ZT5, but not at ZT8. They were then exposed to a 3-h delay of the light-dark (LD) cycle and tested 7 days post-phase shift at the new time corresponding to ZT5 (PS-ZT5; i.e., ZT8 according to the previous LD schedule). No significant alternation was observed at this new circadian phase (i.e., did not differ significantly from chance performance which is 50%). These data suggest that the rhythm in cognition did not phase-shift along with the LD cycle, but that the animals were entrained to the SF interval. (B, top panel) Improvement in spontaneous alternation performance was also not due to modulation by the LD cycle because hamsters performed just as well after 72 h in constant darkness (ZT5 DD), as they did within the LD cycle (ZT5). There were no changes in T-maze arm entries across the various conditions (A, B left middle panel, one-way repeated measures ANOVA: F_(2,11)_=0.81, P=0.456; A, B right middle panel, t-test: P=0.832). Likewise, none of the animals exhibited a bias towards the left or right sides of the T-maze, as none of the positional bias scores deviated significantly from 50% (P>0.05; A,B bottom panels). For alternation scores: * indicates P<0.001.

**
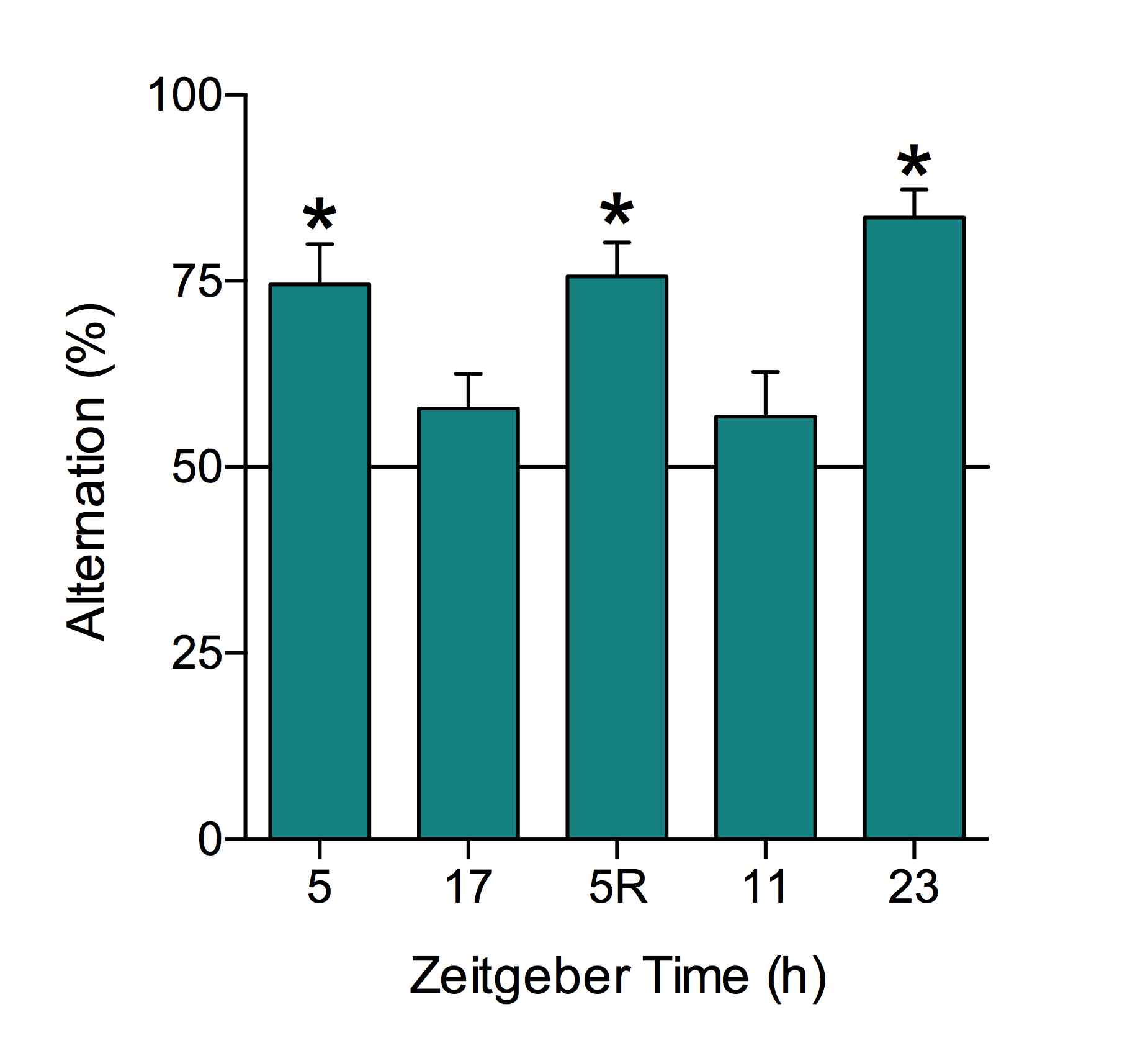
**

**Supplementary Figure 2. Rescue of memory in DPS-arrhythmic hamsters is not a byproduct of the testing sequence.** These are the same data depicted in **Figure 2H**, but plotted left-to-right according to the order in which the animals were tested. This single group of DPS hamsters (n=10) was evaluated at ZT5, 14 days after termination of SF. Subsequent tests at other ZTs were carried out at intervals of 2-4 days. The designation 5R indicates repeat testing at ZT5. For alternation scores: * indicates P<0.001.

**Table 1. Arm entries and positional bias scores for SA tests from Figure 2.**

**Entries (#):**

Panel Group ZT Entries n P

C ENT 5 7.3 ± 0.8 10 0.47 (t-test)

D ARR 5 8.8 ± 0.8 10

G ENT 17 5.6 ± 0.4 10 0.21 (one-way ANOVA, repeated measures)

23 7.1 ± 0.5

5 6.0 ± 0.4

11 5.4 ± 0.4

H ARR 17 6.8 ± 0.5 10 0.58 (one-way ANOVA, repeated measures)

23 6.0 ± 0.4

5 6.2 ± 0.5

11 6.1 ± 0.4

**Positional Bias (%):**

Panel Group ZT PB n P^1^

C ENT 5 60.0 ± 3.1 10 0.14

D ARR 5 48.0 ± 3.0 10 0.40

G ENT 17 47.0 ± 2.8 10 0.39

23 48.0 ± 3.3 0.62

5 45.0 ± 2.4 0.13

11 55.0 ± 5.5 0.41

H ARR 17 43.0 ± 2.4 10 0.13

23 48.0 ± 3.2 0.48

5 49.0 ± 4.0 0.74

11 43.0 ± 3.2 0.18

^1^For positional bias scores, significance was determined by a one-sample t-test against a hypothetical mean of 50%, which indicates no left or right preference in the T-maze. ZT=zeitgeber time, n=sample size.

**Spontaneous Alternation**

Spontaneous alternation (SA) behavior has undergone a resurgence recently as neuroscientists have rediscovered its value in detecting deficits in spatial memory (Deacon and Rawlins, 2006). Nevertheless, many scientists, even those who study behavior, may be unaware that this test is extremely sensitive to hippocampal dysfunction, even more so than the Morris water maze in some cases (Reisel et al, 2002). This discussion summarizes the literature in this area to explain why SA behavior: 1) is specifically dependent on the septohippocampal pathway, 2) allows for frequent re-testing of individual animals without decrements in performance (Douglas, 1989b), 3) does not produce arousal signals as does fear conditioning or require a food reward, both of which would interfere with cognitive performance in our study, and 4) does not require daily training sessions that might stimulate the resurgence of rhythms in arrhythmic hamsters.

SA behavior was first observed in rats over 100 years ago (Hunter, 1914). Since that time, numerous studies have found that organisms as diverse as insects, crustaceans, fish, reptiles, birds, rodents, and humans exhibit alternation behavior (Hughes, 1989). This behavior is thought to form the basis for exploration through a search field that underlies the creation of a cognitive map. Animals are intrinsically motivated to keep track of information relating to the location of food, predators, and potential mates. When traveling through an unfamiliar environment, a species-specific pattern of behavior emerges that serves to orient the animal to its new surroundings and the spatial layout of available resources (Barnett, 1958; Berlyne, 1966). These behaviors, collectively termed *exploration*, occur across the entire search field through which the animal moves and ultimately result in the creation of a cognitive map, which is a complex internal representation of the environment’s physical space (O'Keefe & Nadel, 1978, 1979; Tolman, 1948). Embedded in a cognitive map is the compression of information related to the location of conspecifics, foraging grounds, the relative positioning of three-dimensional objects, routes of escape from predators, reinforcement, and predictions about environmental change (Kaplan, 1973).

Animals continue to update their spatial representations of places over time, maintaining the accuracy of these representations via regular bouts of patrolling (Nadel, 1990). If the environment they are patrolling is given a specific spatial framework, such as a T-maze, they will travel systematically from one endpoint of the apparatus to the next about two-thirds of the time or higher (Gerlai, 1998; Tolman, 1925). The tendency of animals to vary the parts of a T-maze they explore has historically been labeled spontaneous alternation (SA; Dember & Fowler, 1958; Lalonde, 2002). In addition to cognitive maps (O'Keefe & Nadel, 1979), the phenomenon requires a form of spatial working memory that monitors the arm of the maze the animal has been to most recently from the one that it visited on its prior excursion through the maze (Douglas, 1967; Lalonde et al., 1986).

**Role of the Hippocampus in Spontaneous Alternation**

SA is rooted in mammalian brain structures that serve as the substrate for encoding animals’ location in space and processing of novel stimuli. The most important of these structures is the hippocampus. The developmental emergence of SA behavior and of hippocampal maturation occur in parallel in several species (Blair et al., 2013; Bronstein et al., 1974; Douglas et al., 1973; Dumas, 2004; Frederickson & Frederickson, 1979), including humans (Vecera et al., 1991). The latest developing subfield of the hippocampus is the dentate gyrus (DG); the granule cells there (*fascia dentata*) still divide after birth in many species to sculpt a portal of information flow from the medial entorhinal cortex (MEC)—another component of the brain’s navigation system—to the hippocampus. In the rat, SA rates increase as the dentate matures (Douglas et al., 1973). The achievement of adult-like SA performance at the end of the third postnatal week coincides with increases in the synaptic strength of the MEC-DG perforant path (Dumas, 2004). Following along the trisynaptic circuit of the hippocampus, SA in the rat is also associated with the development of excitatory synaptic transmission in the Schaffer collateral pathway between CA3 and CA1 (Blair et al., 2013). Artificial stimulation of this pathway with a positive AMPA receptor modulator accelerates the development of SA by a week (*ibid*).

The close relation between hippocampal maturation and SA behavior in all species studied to date has led many authors to suggest that SA rate is a robust “behavioral marker” of hippocampal integrity (e.g., Blair et al., 2013; Deacon & Rawlins, 2006). Consistent with this premise, *dozens* of lesion studies have shown that adult animals cannot alternate (or exhibit genuine exploration) without an intact hippocampus or MEC inputs (please see O'Keefe & Nadel, 1978, Chapter 6/Table A15, and Douglas, 1989a, for a comprehensive review of this classic lesion work; in particular, note Deacon et al., 2002; Gibbs, 1987; Isseroff, 1979; Johnson et al., 1977; Kimble, 1976, 1978; Kimble and Greene, 1968; Leaton, 1965; Roberts et al., 1962; and Scheff & Cotman, 1977).

By contrast, many other brain regions are expendable for SA; for example, bilateral lesions of the frontal or posterior neocortex, cingulate gyrus, or amygdala do not influence rates of SA in the T-maze (Douglas, 1989). Because SA behavior reliably detects hippocampal performance, the SA task has been used in many seminal studies to characterize the effects on the hippocampus of processes such as aging (Barnes, 1979), Alzheimer’s disease (Holcomb et al., 1998; Hsiao et al., 1996), intellectual disability (Fernandez et al., 2007), or altered glutamate receptor signaling (Reisel et al., 2002; Schmitt et al., 2005). In that regard, it has been shown to be more sensitive to hippocampal dysfunction than the Morris water maze (Reisel et al., 2002).

**Role of the Medial Septum in Spontaneous Alternation**

A synthesis of work done over the past five decades suggests that cholinergic input from the medial septum to the hippocampus is critical for maintaining this function in a T-maze with a continuous trials procedure. Rodents with electrolytic or excitotoxic lesions of the medial septum do not alternate significantly above a chance rate of 50% (Brito & Thomas, 1981; Chang & Gold, 2004; Clody & Carlton, 1969; Dalland, 1970; Douglas & Raphelson, 1966; Hepler et al., 1985; Johnson et al., 1977; Thomas, 1979). Animals treated systemically with anticholinergic drugs, such as scopolamine, also perform poorly in a T or Y-maze task (Douglas & Truncer, 1976; Kokkinidis & Anisman, 1976; Leaton & Utell, 1970; McNaughton & Feldon, 1980; Squire, 1969; for a comprehensive review, see Klinkenberg & Blokland, 2010). These data—collated from adults—complement studies from developing rat pups, which show that SA behavior comes online along the same time-course with which the maturing hippocampus is innervated by the septum (Egger, 1973; Hess & Blozovski, 1987; Kirkby, 1967).

Presumably, the role of the medial septum in SA is derived from its inhabitant population of pacemaker cells, which are necessary to control the frequency of hippocampal theta oscillations generated while an animal moves through space (Stumpf, 1965; Vanderwolf et al., 1973). Septal coordination of theta rhythms, via timed modulation of activity in the EC and hippocampus, regulates arousal and attentional facilities that help juggle short-term representations in working memory (Vanderwolf et al., 1973). Research by the Gold lab suggests that the septohippocampal pathway is highly active during SA and is taxed more when SA behavior is tested in mazes with more arms. Rats exhibit significant spikes in hippocampal acetylcholine release during SA in a four-arm plus maze that is sustained throughout the testing period (a 50-70% increase relative to baseline as determined by *in vivo* microdialysis; Ragozzino & Gold, 1995; Ragozzino et al., 1994, 1996, 1998; Stefani & Gold, 2001).

In summary, the septohippocampal pathway is thought to be the critical structure for expression of alternation behavior because: 1) there is a close association between maturation of septal inputs to the dentate gyrus, and development of the dentate itself, that is necessary for SA behavior, 2) only brain lesions that destroy or alter the circuits associated with this pathway impair SA behavior, whereas damage to several other brain regions outside of the hippocampal system do not, and 3) pharmacological inhibition of septal inputs to the dentate impair SA behavior.

It is important to note that lesions that destroy other brain regions such as the basal forebrain, basal ganglia, raphe system, and amygdala, can impair SA behavior, but those impairments are likely influenced by other factors (Lalonde, 2002). Damage to these areas can confound the SA test by changing the animals’ fear or anxiety state and, in the case of the basal ganglia, can impair the animals’ ability to move (Lalonde, 2002). Lesions of the amygdala also impair SA behavior because of increased anxiety, however, several days of gentle handling reduces anxiety and restores normal SA behavior (Douglas, 1989a).

**Use of Spontaneous Alternation as a Readout of Hippocampal Memory in Food-Entrained Siberian Hamsters**

The aforementioned literature on animal exploration, and the role of the hippocampus and medial septum in this exploration, indicate that the septohippocampus pathway is a circuit that makes especially strong contributions to SA behavior. As such, we used SA as a one-trial procedure to measure hippocampal memory in arrhythmic Siberian hamsters left on *ab libitum* feeding or exposed to a food schedule. Use of this task allowed us to: 1) re-test the hamsters several times to show that a rhythm in memory performance could be elicited within individual cohorts of animals, 2) avoid introducing a competing arousal signal that would interfere with the establishment of food-entrained memory rhythms, and 3) avoid daily training sessions that might stimulate their own rhythm in arrhythmic animals, curbing the expression of the food-entrained rhythm. These goals could not have been met with other hippocampal paradigms such as the Morris water maze, which requires a demanding training program; contextual fear conditioning, which produces stress and an acute arousal signal, or a test with food reward, which would obviously interfere with the food entrainment regimen. It should also be noted that Siberian hamsters are a species that can’t be properly tested in the water maze, because they enjoy swimming and don’t find water to be aversive (Ruby et al., 2008).

**References**

Barnes CA. Memory deficits associated with senescence: a neurophysiological and behavioral study in the rat. J Comp Physiol Psychol. 1979 Feb;93(1):74-104.

Barnett SA. Exploratory behaviour. Br J Psychol. 1958 Nov 1;49(4):289-310.

Berlyne DE. Exploration and curiosity. Science. 1966;153(3731):25-33.

Blair MG, Nguyen NN, Albani SH, L'Etoile MM, Andrawis MM, Owen LM, Oliveira

RF, Johnson MW, Purvis DL, Sanders EM, Stoneham ET, Xu H, Dumas TC. Developmental changes in structural and functional properties of hippocampal AMPARs parallels the emergence of deliberative spatial navigation in juvenile rats. J Neurosci. 2013 Jul 24;33(30):12218-28.

Brito GN, Thomas GJ. T-maze alternation, response patterning, and septo-hippocampal circuitry in rats. Behav Brain Res. 1981 Nov 1;3(3):319-40.

Bronstein PM, Dworkin T, Bilder BH. Age-related differences in rats’ spontaneous alternation. Anim Learn Behav. 1974 Dec 1;2(4):285-8.

Chang Q, Gold PE. Impaired and spared cholinergic functions in the hippocampus after lesions of the medial septum/vertical limb of the diagonal band with 192 IgG-saporin. Hippocampus. 2004;14(2):170-9.

Clody DE, Carlton PL. Behavioral effects of lesions of the medial septum of rats. J Comp Physiol Psychol. 1969 Mar;67(3):344-51.

Dalland T. Response and stimulus perseveration in rats with septal and dorsal hippocampal lesions. J Comp Physiol Psychol. 1970 Apr;71(1):114.

Deacon RM, Bannerman DM, Kirby BP, Croucher A, Rawlins JN. Effects of cytotoxic hippocampal lesions in mice on a cognitive test battery. Behav Brain Res. 2002 Jun 15;133(1):57-68.

Deacon RM, Rawlins JN. T-maze alternation in the rodent. Nat Protoc. 2006;1(1):7-12.

Dember WN, Fowler H. Spontaneous alternation behavior. Psychological Bulletin. 1958 Nov;55(6):412-28.

Douglas RJ. The hippocampus and behavior. Psychological Bulletin. 1967 Jun;67(6):416-42.

Douglas, R. J. (1989a). Spontaneous alternation behavior and the brain. In Spontaneous Alternation Behavior (eds. W. N. Dember & C. L. Richman) pp. 73–108. Springer, New York.

Douglas, R. J. (1989b). Using SAB as a tool: advice from a veteran. In Spontaneous Alternation Behavior (eds. W. N. Dember & C. L. Richman) pp. 145-160. Springer, New York.

Douglas RJ, Peterson JJ, Douglas DP. The ontogeny of a hippocampus-dependent response in two rodent species. Behav Biol. 1973 Jan;8(1):27-37.

Douglas RJ, Raphelson AC. Spontaneous alternation and septal lesions. J Comp Physiol Psychol. 1966 Oct;62(2):320-2.

Douglas RJ, Truncer PC. Parallel but independent effects of pentobarbital and scopolamine on hippocampus-related behavior. Behav Biol. 1976 Nov;18(3):359-67.

Dumas TC. Early eyelid opening enhances spontaneous alternation and accelerates the development of perforant path synaptic strength in the hippocampus of juvenile rats. Dev Psychobiol. 2004 Jul;45(1):1-9.

Egger GJ, Livesey PJ, Dawson RG. Ontogenetic aspects of central cholinergic involvement in spontaneous alternation behavior. Dev Psychobiol. 1973 Jul;6(4):289-99.

Fernandez F, Morishita W, Zuniga E, Nguyen J, Blank M, Malenka RC, Garner CC. Pharmacotherapy for cognitive impairment in a mouse model of Down syndrome. Nat Neurosci. 2007 Apr;10(4):411-3.

Frederickson CJ, Frederickson MH. Emergence of spontaneous alternation in the kitten. Dev Psychobiol. 1979 Nov;12(6):615-21.

Gerlai R. A new continuous alternation task in T-maze detects hippocampal dysfunction in mice. A strain comparison and lesion study. Behav Brain Res. 1998 Sep;95(1):91-101.

Gibbs RB, Yu J, Cotman CW. Entorhinal transplants and spatial memory abilities in rats. Behav Brain Res. 1987 Oct;26(1):29-35.

Hepler DJ, Olton DS, Wenk GL, Coyle JT. Lesions in nucleus basalis magnocellularis and medial septal area of rats produce qualitatively similar memory impairments. J Neurosci. 1985 Apr 1;5(4):866-73.

Hess C, Blozovski D. Hippocampal muscarinic cholinergic mediation of spontaneous alternation and fear in the developing rat. Behav Brain Res. 1987 Jun;24(3):203-14.

Holcomb L, Gordon MN, McGowan E, Yu X, Benkovic S, Jantzen P, Wright K, Saad I, Mueller R, Morgan D, Sanders S, Zehr C, O'Campo K, Hardy J, Prada CM, Eckman C, Younkin S, Hsiao K, Duff K. Accelerated Alzheimer-type phenotype in transgenic mice carrying both mutant amyloid precursor protein and presenilin 1 transgenes. Nat Med. 1998 Jan;4(1):97-100.

Hsiao K, Chapman P, Nilsen S, Eckman C, Harigaya Y, Younkin S, Yang F, Cole G. Correlative memory deficits, Abeta elevation, and amyloid plaques in transgenic mice. Science. 1996 Oct 4;274(5284):99-102.

Hughes RN (1989) Phylogenetic comparisons. In Spontaneous Alternation Behavior (eds. W. N. Dember & C. L. Richman) pp. 39-58. Springer, New York.

Hunter WS (1914) The auditory sensitivity of the white rat. J Animal Behav. 4: 215-222.

Isseroff A. Limited recovery of spontaneous alternation after extensive hippocampal damage: evidence for a memory impairment. Exp Neurol. 1979 May;64(2):284-94.

Johnson CT, Olton DS, Gage FH 3rd, Jenko PG. Damage to hippocampus and hippocampal connections: effects on DRL and spontaneous alternation. J Comp Physiol Psychol. 1977 Jun;91(3):508-22.

Kaplan, S. (1973). Cognitive maps, human needs and the designed environment. In Environmental Design Research, vol. 1 (ed. W. F. E. Preiser), pp. 275–283. Dowden, Hutchinson, and Ross, Stroudsburg, Pa.

Kimble DP. Changes in behavior of hippocampal-lesioned rats across a 6-week postoperative period. Physiol Psychol. 1976 Sep;4(3):289-93.

Kimble DP. Effects of combined entorhinal cortex-hippocampal lesions on locomotor behavior, spontaneous alternation and spatial maze learning in the rat. Physiol Behav. 1978 Aug;21(2):177-87.

Kimble DP, Greene EG. Absence of latent learning in rats with hippocampal lesions. Psychonomic Science. 1968 Mar;11(3):99-100.

Kirkby RJ. A maturation factor in spontaneous alternation. Nature. 1967 Aug;215:784.

Klinkenberg I, Blokland A. The validity of scopolamine as a pharmacological model for cognitive impairment: a review of animal behavioral studies. Neurosci Biobehav Rev. 2010 Jul 31;34(8):1307-50.

Kokkinidis L, Anisman H. Interaction between cholinergic and catecholaminergic agents in a spontaneous alternation task. Psychopharmacology (Berl). 1976 Aug 17;48(3):261-70.

Lalonde R. The neurobiological basis of spontaneous alternation. Neurosci Biobehav Rev. 2002 Jan;26(1):91-104.

Lalonde R, Botez MI, Boivin D. Spontaneous alternation and habituation in a t-maze in nervous mutant mice. Behav Neurosci. 1986 Jun;100(3):350-2.

Leaton RN. Exploratory behavior in rats with hippocampal lesions. J Comp Physiol Psychol. 1965 Jun;59:325-30.

Leaton RN, Utell MJ. Effects of scopolamine on spontaneous alternation following free and forced trials. Physiol Behav. 1970 Mar 31;5(3):331-4.

McNaughton N, Feldon J. Spontaneous alternation of body turns and place: differential effects of amylobarbitone, scopolamine and septal lesions. Psychopharmacology (Berl). 1980;68(2):201-6.

Nadel L. Varieties of spatial cognition. Ann N Y Acad Sci. 1990 Dec 1;608(1):613-36.

McNay EC, Fries TM, Gold PE. Decreases in rat extracellular hippocampal glucose concentration associated with cognitive demand during a spatial task. Proc Natl Acad Sci U S A. 2000 Mar 14;97(6):2881-5.

O'keefe, J., & Nadel, L. (1978). The hippocampus as a cognitive map. Oxford University Press, USA.

O'Keefe J, Nadel L. Precis of O'Keefe & Nadel's The hippocampus as a cognitive map. Behav Brain Sci. 1979 Dec 1;2(04):487-94.

Ragozzino ME, Gold PE. Glucose injections into the medial septum reverse the effects of intraseptal morphine infusions on hippocampal acetylcholine output and memory. Neuroscience. 1995 Oct 31;68(4):981-8.

Ragozzino ME, Pal SN, Unick K, Stefani MR, Gold PE. Modulation of hippocampal acetylcholine release and spontaneous alternation scores by intrahippocampal glucose injections. J Neurosci. 1998 Feb 15;18(4):1595-601.

Ragozzino ME, Unick KE, Gold PE. Hippocampal acetylcholine release during memory testing in rats: augmentation by glucose. Proc Natl Acad Sci U S A. 1996 May 14;93(10):4693-8.

Ragozzino ME, Wenk GL, Gold PE. Glucose attenuates a morphine-induced decrease in hippocampal acetylcholine output: an in vivo microdialysis study in rats. Brain Res. 1994 Aug 29;655(1-2):77-82.

Reisel D, Bannerman DM, Schmitt WB, Deacon RM, Flint J, Borchardt T, Seeburg PH, Rawlins JN. Spatial memory dissociations in mice lacking GluR1. Nat Neurosci. 2002 Sep;5(9):868-73.

Roberts WW, Dember WN, Brodwick M. Alternation and exploration in rats with hippocampal lesions. J Comp Physiol Psychol. 1962 Oct;55:695-700.

Ruby NF, Hwang CE, Wessells C, Fernandez F, Zhang P, Sapolsky R, Heller HC.

Hippocampal-dependent learning requires a functional circadian system. Proc Natl

Acad Sci U S A. 2008 Oct 7;105(40):15593-8.

Scheff SW, Cotman CW. Recovery of spontaneous alternation following lesions of the entorhinal cortex in adult rats: possible correlation to axon sprouting. Behav Biol. 1977 Oct;21(2):286-93.

Schmitt WB, Sprengel R, Mack V, Draft RW, Seeburg PH, Deacon RM, Rawlins JN, Bannerman DM. Restoration of spatial working memory by genetic rescue of GluR-A-deficient mice. Nat Neurosci. 2005 Mar;8(3):270-2.

Squire LR. Effects of pretrial and posttrial administration of cholinergic and anticholinergic drugs on spontaneous alternation. J Comp Physiol Psychol. 1969 Sep;69(1):69-75.

Stefani MR, Gold PE. Intrahippocampal infusions of k-atp channel modulators influence spontaneous alternation performance: relationships to acetylcholine release in the hippocampus. J Neurosci. 2001 Jan 15;21(2):609-14.

Stumpf C. Drug action on the electrical activity of the hippocampus. Int Rev Neurobiol. 1965;8:77-137.

Thomas GJ. Comparison of effects of small lesions in posterodorsal septum on spontaneous and rerun correction (contingently reinforced) alternation in rats. J Comp Physiol Psychol. 1979 Aug;93(4):685-694.

Tolman EC. Purpose and cognition: the determiners of animal learning. Psychol Rev. 1925; 32:285–97.

Tolman EC. Cognitive maps in rats and men. Psych. Rev. 1948;55,189-208.

Vanderwolf, C. H., Bland, B. H., and Whishaw, I. Q. (1973). Diencephalic hippocampal and neocortical mechanisms in voluntary movement. In Efferent Organization and the Integration of Behaviour (ed. J. D. Maser) pp. 229–262. Academic Press, New York.

Vecera SP, Rothbart MK, Posner MI. Development of spontaneous alternation in

infancy. J Cogn Neurosci. 1991 Fall;3(4):351-4.
